# Temperature Distribution in TaO_*x*_ Resistive Switching Devices Assessed In Operando by Scanning Thermal Microscopy

**DOI:** 10.1021/acsaelm.3c00229

**Published:** 2023-04-10

**Authors:** Jingjia Meng, Jonathan M. Goodwill, Evgheni Strelcov, Kefei Bao, Jabez J. McClelland, Marek Skowronski

**Affiliations:** † Department of Materials Science and Engineering, Carnegie Mellon University, Pittsburgh, Pennsylvania 15213, United States; ‡ Physical Measurement Laboratory, National Institute of Standards and Technology, Gaithersburg, Maryland 20899, United States; § Department of Chemistry and Biochemistry, University of Maryland, College Park, Maryland 20742, United States

**Keywords:** ReRAM, scanning thermal microscopy, electroformation, filament, memory switching, tantalum oxide

## Abstract

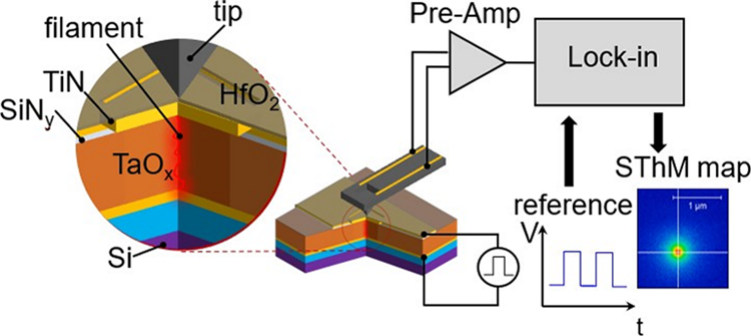

Understanding the physical changes during electroformation and switching processes in transition-metal-oxide-based non-volatile memory devices is important for advancing this technology. Relatively few characteristics of these devices have been assessed in operando. In this work, we present scanning thermal microscopy measurements in vacuum on TaO_*x*_-based memory devices electroformed in both positive and negative polarities and high- and low-resistance states. The observed surface temperature footprints of the filament showed higher peak temperatures and narrower temperature distributions when the top electrode served as the anode in the electroformation process. This is consistent with a model in which a hot spot is created by a gap in the conducting filament that forms closest to the anode. A similar behavior was seen on comparing the high-resistance state to the low-resistance state, with the low-resistance footprint showing a lower peak and a larger width, consistent with the gap disappearing when the device is switched from high resistance to low resistance.

## Introduction

Oxide-based non-volatile memory devices are explored for applications such as embedded memory^1^ and in neuromorphic computing schemes.^2,3^ Owing to their advantages such as back-end-of-line compatibility, fast switching speed, and multilevel resistive switching, the oxides show promise in meeting the demands for increased storage density and computing speed.^4^ Since the first commercialized oxide-based memory product was introduced in 2013, TaO_*x*_-based resistive random access memory (ReRAM) has been used to replace embedded SRAM in a 22 nm process.^5,6^ Also, several foundries have incorporated ReRAM into their process design kits.^7^ While this is certainly promising and allows for the design and fabrication of novel circuits, the endurance of ReRAM devices at 10^4^ cycles is not nearly good enough for use in applications such as compute-in-memory.^8,9^ More research is needed to understand the mechanisms involved in ReRAM operation and to optimize the device design and fabrication processes.

Most of the research efforts in oxide ReRAM have been focused on correlating materials and device design with the electrical characteristics of devices. A few studies, based on transmission electron microscopy (TEM)^10−12^ and X-ray microscopy (XRM),^13−15^ have attempted to image the structure and composition of the electroformed devices, leading to a generally accepted model of the switching involving a conducting filament created during electroformation. The filament has a small gap that opens and closes for the high-resistance (HRS) and low-resistance states (LRS) as a result of oxygen vacancy migration.^16−18^ Both TEM and XRM are limited in sensitivity, however, since they can only detect changes in compositions above about 5 atom %. This detection level is inadequate for studying current distributions, since the electrical conductivity of an oxide changes by many orders of magnitude for dopant concentration or stoichiometry deviations well below 1%.^19^ This leaves a large gap in our understanding of how ReRAM devices operate electrically at the microscopic level since the current density can extend deep into areas that would be considered insulating based on these measurements. Scanning probe microscopy measurements of the temperature distribution on the surface of the device can help fill this gap because they can provide insights into the current density distribution.^20^ The results are expected to verify and complement the analytical data, leading to a fuller picture of the physical processes underlying the switching phenomena.

Scanning thermal microscopy (SThM) images of HfO_2_-based resistive switches using a sharp V-shaped thermoresistor in contact with the sample in air have recently been reported.^21^ The topographical resolution of these measurements was limited to about 100 nm by the size of the tip. The thermal resolution was also further influenced by heat transport through the air and water meniscus surrounding the scanning tip, requiring complex deconvolution. These deconvolutions were necessary because the thermal distribution widths on the sample surface are expected to also be in the range of 100 nm due to thermal spreading during heat transport from the hot zone in the filament to the surface. The present study does not have these additional complications because the experiments were conducted in vacuum. However, the thermal spreading in the sample still makes it difficult to probe the filament which, as the TEM images suggest, is of the order 10–20 nm in size.^10,22^

Despite the effects of heat spreading in the sample, SThM can still provide valuable information about the filament and the switching mechanism in ReRAM devices by careful measurement of the temperature footprint on the surface.^23^ Modeling of the switching process predicts that the highest temperature will occur in the gap region due to its lower conductivity.^16,24^ Because of heat spreading, we expect the position of the hot spot along the filament to have an influence on the size of the thermal footprint on the surface: the closer the hot spot is to the surface, the narrower the width and the higher the amplitude of the distribution. This correlation between thermal footprint width and hot spot location can be used to interpret SThM measurements, allowing for the determination of the gap location as a function of device state and formation polarity. The elucidation of temperature distribution and its maximum is critical for understanding device failure, as most failure mechanisms are thermally activated.

In this work, we present SThM measurements of the temperature distribution on the top surface of symmetric TiN/TaO_*x*_/TiN-basedresistive switching devices. Experiments were conducted in vacuum using a scanning probe microscope system integrated into a scanning electron microscope (SEM). We compare two nominally identical devices electroformed in opposite polarities. Devices formed with the top electrode as an anode are expected to form a gap in the filament at the top of the oxide near the interface. Similarly, devices formed with the bottom electrode as an anode would have a gap near the bottom of the oxide.^11^ We also compare the temperature distribution of a device in the HRS and LRS, observing changes in the gap caused by switching between these two states.

## Experimental Method

**Figure 1:**
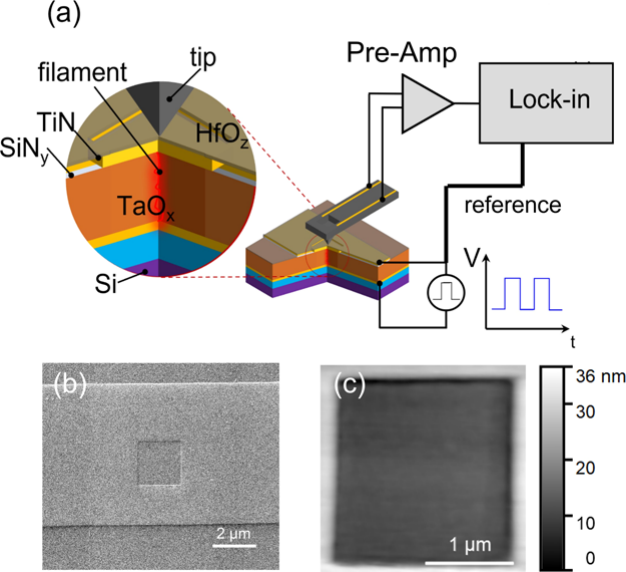
(a) Experimental setup of the SThM measurements and the device structure. (b) An SEM image showing the entire area of the crossbar region. (c) Topography scan of the via using the SThM tip.

The device structure used in this study consisted of a TiN/TaO_*x*_/TiN sandwich, with the initial composition of the functional layer being approximately TaO_2_.^19^ The structure was nominally symmetric, although some asymmetry was unavoidable due to heat sinking differences at the top and bottom interfaces and the patterning process of the top electrode, which exposed the TaO_*x*_/TiN interface to solvents. The geometry was that of an inverted via as shown in Figure 1a. A large bottom electrode, the functional layer, and a 20 nm thick SiN_*y*_ insulator were sputter-deposited without breaking vacuum. This was followed by reactive ion etching of a 2 μm × 2 μm window in the SiN_*y*_. Finally, a 20 nm thick top TiN electrode and an 8 nm thick HfO_2_ passivation layer were sputter-deposited. These materials exhibit low thermal conductivities, which prevent heat spreading. Also, HfO_2_ provides electrical isolation of the top electrode and metallic thermocouple used as the scanning tip and a smooth surface morphology. The 30 nm thick functional layer was deposited by reactive sputtering with an oxygen partial pressure of 0.020 Pa. Figure 1b is an SEM image of the crossbar area. The bottom electrode was patterned together with a series resistance of 14 kΩ to prevent uncontrolled current spikes during electroformation and switching. Special care was paid to obtain low surface roughness of the structure to ensure a uniform thermal contact between the tip and the surface during SThM imaging. The root-mean-square surface roughness was less than 1.9 nm within the 2 μm × 2 μm window, with just a few protrusions, located mostly close to the edge of the active region. The topography scan of the via taken with the SThM probe is shown in Figure 1c.

The SThM images were collected using a silicon cantilever with a hollow SiO_2_ tip integrated with a thermocouple at the apex.^25^ It is expected that the lateral resolution of this tip for temperature measurements is about 50 nm. The cantilever was held using an adapted tip holder in a commercial scanning probe microscope (SPM) mounted on the stage of a SEM. This system allowed us to carry out SThM measurements in vacuum (≈6 × 10^–4^ Pa), eliminating many of the complications associated with thermal conduction through air and water meniscus at the tip. It also allowed accurate probe placement through observation with the SEM during approach and landing. The thermocouple signal was amplified by a preamplifier with a nominal gain of 100 located in vacuum next to the SPM system to reduce electronic noise. Measurements were conducted by applying a 5 kHz square-wave voltage to the device and detecting the pre-amplified thermal signal with a lock-in amplifier. Due to the small, heated volume, quasi-steady-state conditions were reached in the device in a time much shorter than the 0.1 μs on-time of the square wave.

## Results and Discussion

Devices were initially electroformed by applying a series of increasing quasi-static voltage sweeps starting at 0 V and ending as high as 13 V (here, we refer to the source voltage; the device voltage was smaller due to the voltage drop across the series resistor, which depended on the current in the device). The amplitude of the sweep was increased until a permanent change in device resistance was detected. Current compliance was set to 450 μA to limit the maximum current flow through the device. Positive bias is defined here as a positive voltage on the top electrode.

**Figure 2:**
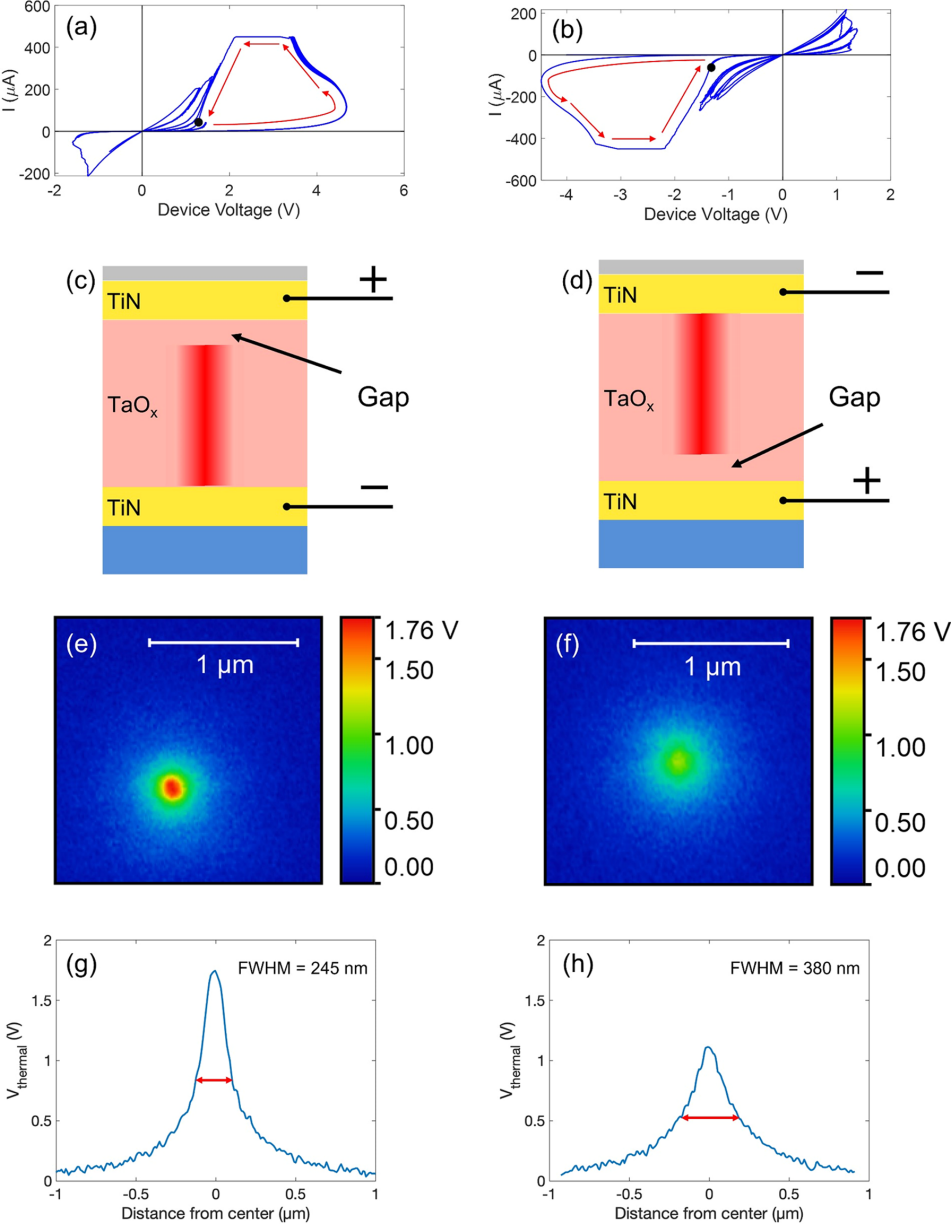
(a,c,e,g) Device formed in positive polarity. (a) Voltage sweeps leading to electroformation and subsequent *I*–*V* switching characteristics. The black dot marks the operating point of the SThM scan. (c) Schematic drawing of the conducting filament. (e) SThM surface temperature map. (g) Temperature line profile extracted from the 2D scan across the center of the hot spot. (b,d,f,h) Forming and switching *I*–*V*, filament schematics, temperature map, and temperature line profile of the device electroformed in negative polarity.

Figure 2a shows the *I*–*V* characteristics of a device electroformed with positive polarity. The measured current is plotted as a function of the voltage across the device, where we have subtracted the voltage drop across the 14 kΩ series resistor from the measured source voltage. During the initial sweeps with lower maximum voltage, the *I*–*V* curves show the typical S-shape of threshold switching devices in the as-fabricated state.^26^ As the maximum voltage is increased with each sweep, at a point while the device is in the negative differential resistance region, the current spontaneously increases dramatically, reaching the compliance limit and causing a large voltage drop across the series resistor (red arrows in the figure). After this event, which corresponds to the formation of the conducting filament, the device *I*–*V* curve becomes permanently altered, taking on the characteristic hysteresis of a resistive switching device. As discussed in previous reports, electroformation in TiN/TaO_*x*_/TiN structures is thought to be caused by a large temperature gradient that develops as the result of current constriction in the negative differential resistance part of *I*–*V*.^26^ This gradient drives Ta ions toward the center of the hot spot and O ions away from it, creating a Ta-rich and O-poor conducting cylinder.^19,27,28^ The process is self-accelerating, resulting in a compositional runaway and collapse of the initial current constriction of about 1 μm diameter into a nanometer-size permanent filament.^24^ This forming process leaves the device in the HRS with the gap located at the interface between the top electrode and the functional layer (Figure 2c). Figure 2b,d shows the *I*–*V* curve for the second device, formed in negative polarity, and the filament configuration. The *I*–*V* characteristics are similar to the positive polarity case, with formation occurring at *V*_DEVICE_ = −3.5 V and a dissipated power of 1575 μW.

Temperature scans on electroformed devices were performed using a square wave with a source voltage amplitude of 2 V. The corresponding device voltage and current are marked by the black dots in Figure 2a,b (*V*_DEVICE_ = 1.23 V, *I* = 59 μA, and dissipated power of 73 μW). The resulting surface temperature maps (Figure 2e,f) show a clear hot spot corresponding to a filament located close to the center of the device.

The temperature maps in Figure 2e,f are expressed in terms of the output voltage from the lock-in amplifier. While it is tempting to perform a calibration of this signal to obtain the surface temperature of the device, there are several factors that make this difficult. The thermal signal from the SThM tip originates from the temperature of the thermocouple junction, which is located a few nanometers away from the actual tip–surface contact. Modeling suggests that a strong thermal gradient exists in this region of the tip; therefore, it cannot be assumed that the thermocouple signal itself represents the surface temperature. In addition, there likely is non-negligible thermal resistance at the tip–surface contact, adding additional uncertainty. Furthermore, modeling suggests that the tip contact on the surface introduces a significant localized heat sinking effect, reducing the surface temperature. According to the model, this reduced temperature only penetrates a few nanometers into the surface and so does not significantly affect the temperature of the active layer in the device. In a future publication, we plan to discuss the finite element modeling used to draw these conclusions and pursue possibilities for extracting actual surface temperatures through a combination of measurements and modeling. For the present work, it is sufficient to consider the relative temperature measurements because we are most interested in observing changes in the surface temperature distribution. Since we use the same amplifier circuit and keep all circuit parameters the same, the voltage reading from the thermocouple has the same scaling factor in all measurements.

Figure 2e shows the thermal map for the device formed with positive polarity. A line scan through the center of the hot spot (Figure 2g) closely follows a Lorentzian shape. The full width at half maximum (FWHM) of this profile is 245 nm with a voltage amplitude of 1.76 V. All FWHM values reported in this paper have an estimated uncertainty of ±10% arising from variations in the profile taken at different angles. A Lorentzian shape of the distribution is expected if the Ta-deficient gap can be considered as a point heat source. For such a source, the heat flux decays with the square of the distance from the source, and a line profile along the surface would be proportional to (*x*^2^ + *d*^2^)^−1^, where *x* is the coordinate along the surface and *d* is the distance of the source below the surface. Figure 2f,h shows the temperature map and line profile. The hot spot is located close to the center of the active region of the device as before, with a similar Lorentzian shape of the profile. The peak amplitude, in this case, is 1.14 V and the FWHM is 380 nm. The peak amplitude is lower, and the FWHM is larger, than in the positive polarity case, even though the dissipated power was the same for each.

The differences between the temperature distributions for the two polarities are fully consistent with the model developed from TEM measurements conducted on very similar devices.^10,11^ In the positive polarity case, a Ta-depleted gap forms at the interface with the top electrode, with the remaining continuous filament extending to the bottom interface (Figure 2c). In the negative polarity case, this arrangement is reversed. Assuming most of the heat is generated in the gap, the temperature map at the surface should be narrower and with higher maximum when the gap is at the top interface and wider and lower when the gap is at the bottom. This difference is precisely what one can see in the two thermal scans shown in Figure 2.

**Figure 3:**
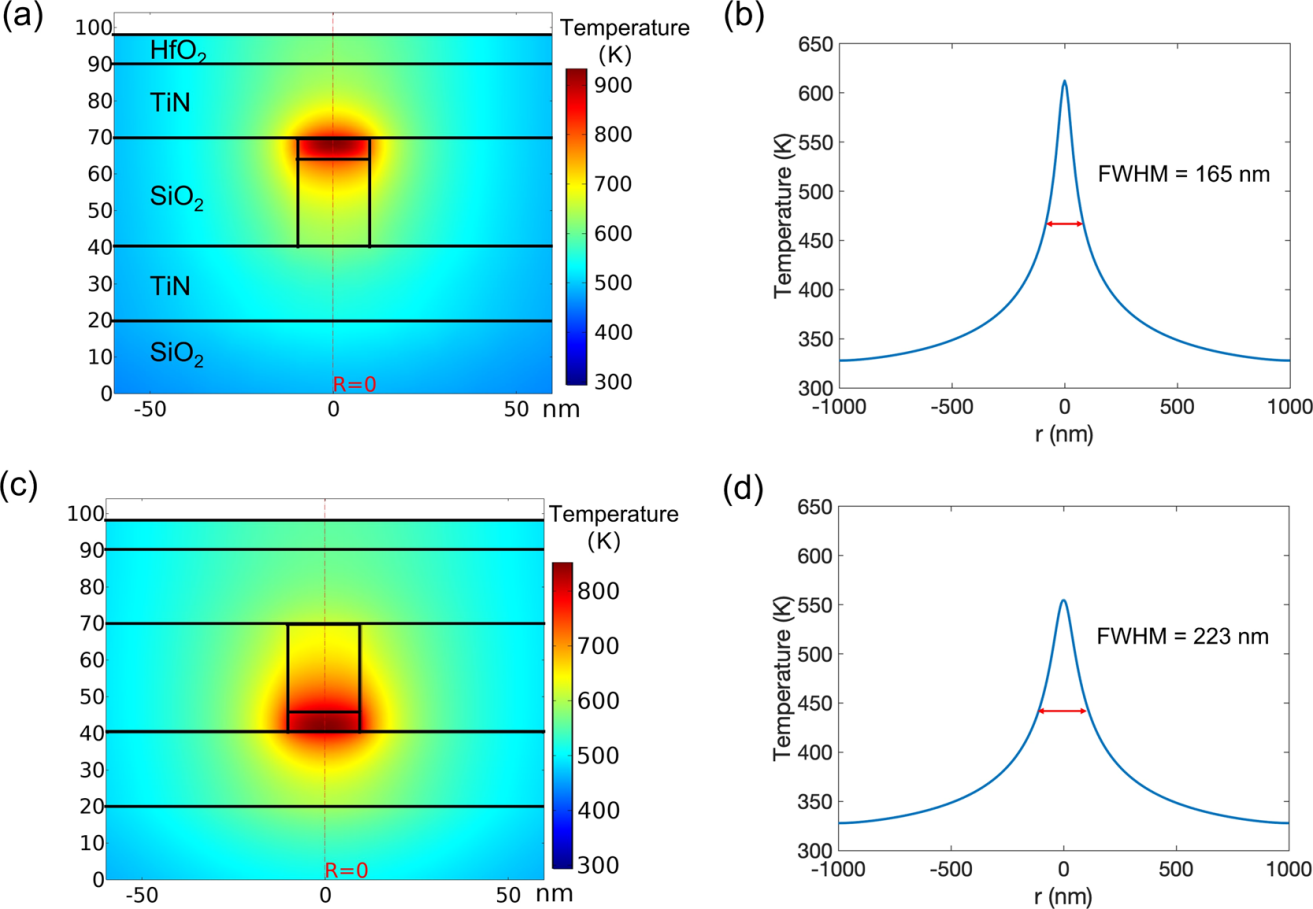
Multiphysics finite element model of temperature distribution on the cross-section (a) and the line profile on the top surface (b) of the device formed and biased in positive polarity. Such a device has the Ta-deficient gap close to the top electrode. (c,d) Corresponding results of the device formed and biased in negative polarity.

To further elucidate the observed temperature distributions in the electroformed devices studied here, we constructed a multiphysics finite element model to solve for the electrical current and heat flow. The model assumes a sandwich metal/insulator/metal structure with the thicknesses of all layers as listed earlier in this report and a cylindrical conducting filament with a diameter of 20 nm that has a 6 nm wide gap located next to the top or bottom electrode. The schematic diagrams of the device structure and the filament are shown as black outlines in Figure 3a,c. The material parameters used in the simulation are listed in Table 1. The electrical conductivities of the filament and the gap were selected to match the experimentally measured device resistance at the operating point.

**Table 1:** Thermal and Electrical Parameters Used in the Simulation

material	density (kg·m^–3^)	thermal conductivity (W·m^–1^·K^–1^)	electrical conductivity (S·m^–1^)	heat capacity (J·kg^–1^·K^–1^)
SiO_2_	2200	1.4	0	730
TiN	5220	5	5 × 10^6^	545
TaO_*x*_ (peripheral)	1 × 10^4^	10	1 × 10^–3^	174
TaO_*x*_ (filament)	1 × 10^4^	25	5 × 10^4^	174
TaO_*x*_ (uniform)	1 × 10^4^	25	2 × 10^4^	174
TaO_*x*_ (gap)	1 × 10^4^	25	1 × 10^3^	174
HfO_*z*_	1 × 10^4^	1.5	0	120

Since the electron density in the TaO_*x*_ is low and the phonon spectra in TaO_*x*_ and TiN are mismatched,^29,30^ one can expect a significant thermal boundary resistance. As the phonon density in TaO_*x*_ is similar to that of MgO, we have estimated such resistance by linear regression fit of the conductivity data of Lyeo and Cahill^31^
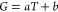
1where *G* is thermal conductance per unit area, *a* = 2.14 MW•K^–2^•m^–2^, and *b* = 94.5 MW•K^–1^•m^–2^. Figure 3a shows the modeled temperature distribution on the cross-section of a device formed in positive polarity with the gap located next to the interface with the top electrode. The hottest spot of the entire device appears in the center of the Ta-deficient gap, with a maximum temperature of 910 K. Figure 3b shows the line profile of the temperature on the top surface. The maximum temperature is located right above the filament with a maximum of 612 K and an FWHM of 165 nm. The FWHM is about an order of magnitude larger than the size of the filament imaged by TEM in nominally identical structures.^10^ This size is determined by, and increases with, the thermal conductivity of the materials used, the thicknesses of the TiN electrode and HfO_2_ passivation, and the thermal boundary resistance. Figure 3c,d shows the corresponding temperature distributions in the device formed in negative polarity. The maximum temperature again appears in the center of the gap, this time located at the interface with the bottom electrode. It is noticeably smaller (855 *vs* 910 K) than that in Figure 3a. The lower peak temperature in the gap is due to the location of the gap being closer to the heat sink at the bottom of the SiO_2_ layer in the modeled slab. Moreover, the gap at the bottom interface implies a larger distance from the top surface. As the thermal resistance increases with the path length, and the temperature drop scales with the thermal resistance, it further reduces the temperature at the top surface. Combining these two factors, the peak temperature on the surface is lower when the gap is deeper in the device. A larger FWHM is also a consequence of the longer thermal path. The maximum temperature at the top surface, 554 K, is lower by 58 K and the FWHM is larger (223 *vs* 165 nm).

While the simulation managed to reproduce the overall temperature characteristics of the two configurations, the calculated FWHMs are approximately 100 nm smaller than the measured values. While we cannot completely rule out tip resolution effects, we believe that this discrepancy comes from the assumption of an abrupt filament composition change and the temperature-independent electrical conductivity of the surrounding oxide. This is a simplification made for ease of modeling. In the actual filament, the composition and the conductivity are expected to change continuously with the distance from the center of the filament.^19^ As a consequence, the current distribution should be wider than the assumed filament with the resulting heat source having a larger diameter.

**Figure 4:**
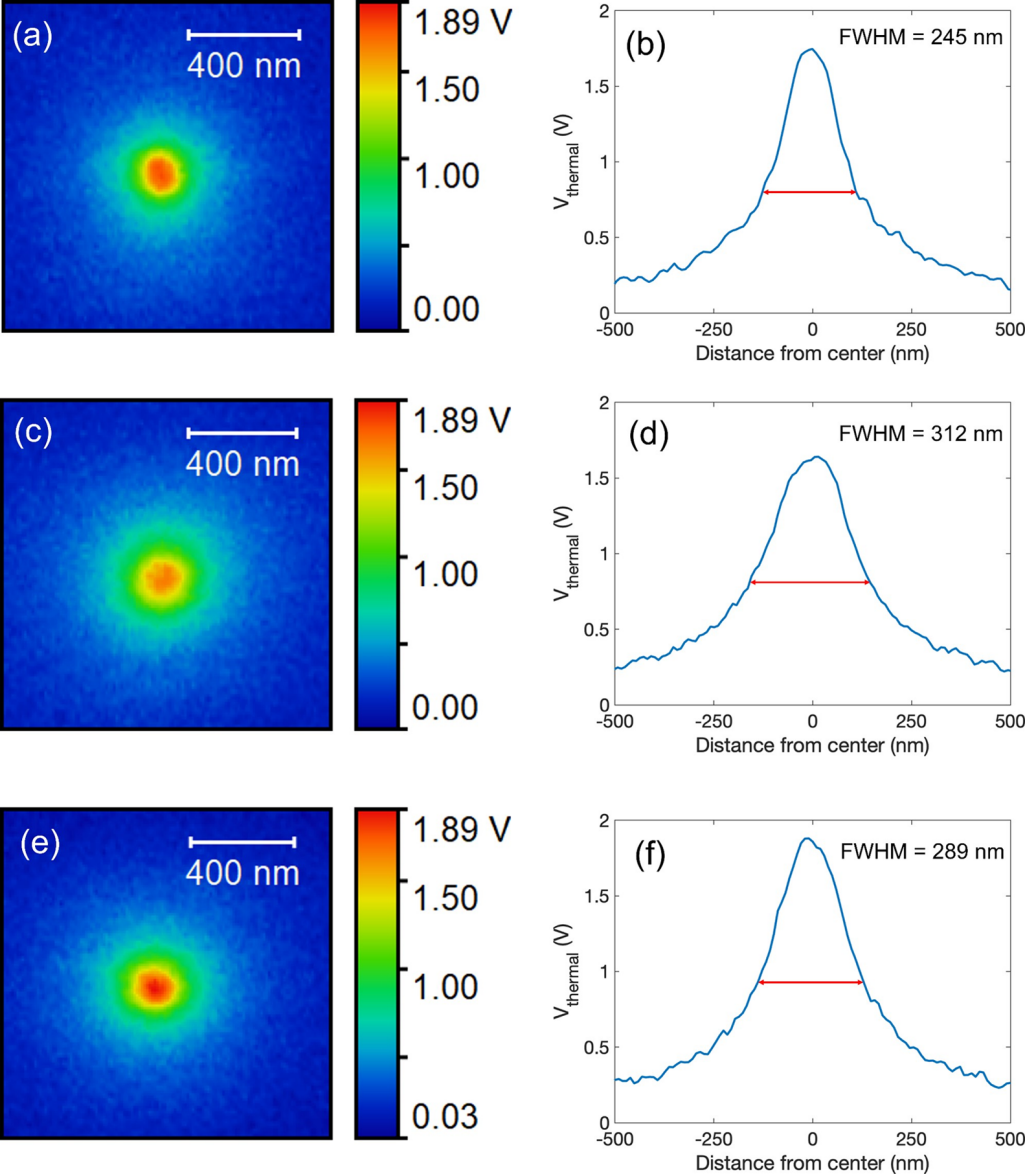
(a,b) Surface temperature map of a just-formed device in HRS and its corresponding temperature line profile across the hottest point. (c,d) Temperature map of the same device after switching to LRS and the corresponding temperature line profile. (e,f) Temperature map and profile after switching to the HRS state. Images (a) and profile (b) are the same as in Figure 2e,g.

Another set of experiments was conducted to examine the temperature distribution differences between the LRS and HRS states. In this set, we compared three temperature maps obtained on the same device (Figure 4). The first, collected on the device that was just electroformed and left in the HRS, is shown in Figure 4a with the corresponding line profile across the center of the hot spot in Figure 4b. These data are the same as those shown in Figure 2e,f. The device voltage during the SThM scan was 1.23 V, the current was 59 μA, and the power dissipation was 73 μW. The highest thermal signal was 1.76 V and the FWHM was 245 nm. The second scan was performed after switching the device to the LRS state using a quasi-DC sweep with a rate of 4 V/s (Figure 4c,d). The temperature scan was collected at almost the same dissipated power of 71 μW (device voltage of 0.73 V and current of 98 μA). The peak thermal signal was 1.64 V, and the FWHM was 312 nm, values lower and wider than those seen in the HRS. Figure 4e,f shows the thermal map after the device was switched back to the HRS, collected with a dissipated power of 74 μW. In this case, the peak thermal signal increased to 1.89 V and the FWHM reduced to 289 nm. Both these values are very close to the values seen in the original, just-electroformed HRS state. The interpretation of these observations parallels the one provided above: the hottest spot in the filament was close to the top TaO_*x*_/TiN interface in images (a) and (e), while it was located lower in the device in image (c). The increased thermal path in (c) results in the changes observed in the experiment.

**Figure 5:**
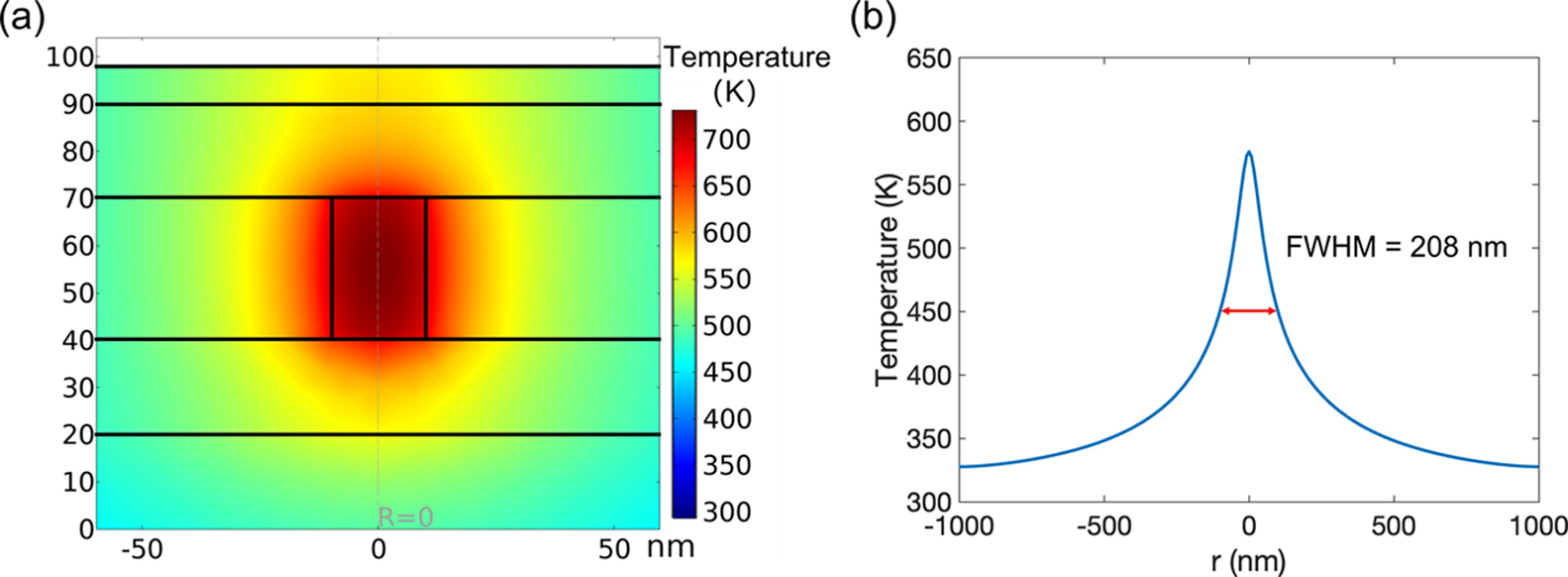
Finite element model of temperature distribution on the cross-section (a) and the line profile on the top surface (b) of the LRS device (a uniform filament).

In order to support this qualitative argument, we used the multiphysics finite element model to simulate the temperature distribution in the LRS, as shown in Figure 5. In this case, the filament was assumed to be a uniform conductive cylinder with a diameter of 20 nm. We kept all parameters the same as in Figure 3a,c, only removing the Ta-depleted gap. The maximum temperature reached was 713 K, around the center of the filament, and the surface profile showed a peak temperature of 576 K and an FWHM of 208 nm. We can see that although the diameter of the filament remained unchanged, the temperature width on the surface increased by 43 nm. This increased size, simply due to the relocation of the hottest point to the middle of the filament column, is comparable to the experimental result for the device switched to LRS.

An alternative interpretation of the increased FWHM is suggested by the TEM images published by Ma et al*.*^12^ According to these authors, the high temperature and the oxygen-rich composition within the filament gap result in the crystallization of stable Ta_2_O_5_. This prevents the reduction of the oxide during the set process. Instead, Ta-rich and O-poor sub-filaments form at the periphery of the gap, increasing its diameter.

## Conclusions

In this work, we used SThM to assess the temperature distribution on the surface of TiN/TaO_*x*_/TiN-based resistive switching devices. In particular, we have measured and analyzed the temperature distributions in proximity of the conducting filaments. By comparing the temperature maps for devices formed in positive and negative polarities, we have confirmed that the gap in the filament is located at the interface of the functional oxide and the electrode that was positively charged during the electroformation process. This result agrees with the TEM data obtained on similar devices. The location of the gap changes between the top and bottom interfaces with the reversal of the electroforming bias. In the HRS, the highest temperature is reached within the resistive gap. In the LRS, the results are consistent with the uniform temperature distribution along the filament. A better understanding of the temperature distribution along the filament offers new insights into the origin of interdiffusion between oxide and the electrodes. The high temperature found within the resistive gap provides a plausible interpretation for the formation of crystalline TaO_*x*_ in failed devices^12^ and provides motivation to increase the high-temperature stability of this system to improve endurance.
